# Value of abdominal ultrasonography in predicting intestinal resection for premature infants with necrotizing enterocolitis

**DOI:** 10.1186/s12876-022-02607-0

**Published:** 2022-12-16

**Authors:** Jingyu Chen, Fangting Mu, Kai Gao, Chengwei Yan, Gongli Chen, Chunbao Guo

**Affiliations:** 1 Department of Ultrasound, Children’s Hospital, Chongqing Medical University, Chongqing, People’s Republic of China; 2 Department of Pediatrics, Women and Children’s Hospital, Chongqing Medical University, 120 Longshan Rd., Chongqing, 401147 People’s Republic of China; 3 Department of Pediatric General Surgery, Chongqing University Three Gorges Hospital, Chongqing, People’s Republic of China; 4 Ministry of Education Key Laboratory of Child Development and Disorders, Chongqing Medical University, Chongqing, People’s Republic of China; 5 Department of Pediatrics, Chongqing Health Center for Women and Children, Chongqing, People’s Republic of China

**Keywords:** Necrotizing enterocolitis, Abdominal ultrasonography, Abdominal radiograph, Intestinal resection

## Abstract

**Background:**

Abdominal ultrasonography (AUS) has been suggested to be valuable in the early detection of necrotizing enterocolitis (NEC).

**Objective:**

Here, we intended to assess the efficiency of abdominal ultrasound in terms of predicting bowel resection in infants with NEC.

**Methods:**

From January 2018 to March 2021, 164 hospitalized children with NEC who underwent surgical management were enrolled. The enrolled infants were separated into two groups according to whether they underwent bowel resection. We reviewed the clinical data, as well as dynamic sonographic and abdominal radiographic (AR) parameters. The potential performance of AUS parameters was identified and compared with that of AR using a logistic regression analysis and receiver operating characteristic (ROC) curve analysis.

**Results:**

Several parameters were detected to be statistically significant in predicting the occurrence of bowel resection, including thick to thin transformation [*p* < 0.001, odds ratios (OR), 4.38; 95% confidence interval (CI) 2.26–8.52], absence of peristalsis certain areas (*p* = 0.001, OR, 2.99; 95% CI 1.53–5.86), absence of bowel wall perfusion (*p* < 0.001, OR 10.56; 95% CI 5.09–21.90), and portal venous gas (*p* = 0.016, OR, 2.21; 95% CI 1.12–4.37). Furthermore, the ROC curve analysis showed significant differences (*p* = 0.0021) in the area under the receiver operating curve (AUROC) for the logistic models of AUS (AUROC: 0.755, 95% CI 0.660–0.849) and AR (AUROC: 0.693, 95% CI 0.597–0.789) for the prediction of intestinal resection.

**Conclusions:**

A dynamic AUS examination play an important role to indicate the potential for bowel loss during the surgery.

## Introduction

Necrotizing enterocolitis (NEC) is the most destructive gastrointestinal disease in preterm infants and usually requires surgical management [1, 2]. The primary clinical manifestations of NEC are often nonspecific, including abdominal distension, vomiting and bloody stool, but the disease can progress rapidly to severe symptoms such as bradycardia, even shock and apnea [3, 4]. The identification of patients who may benefit from surgical intervention to avoid bowel loss is crucial, but has only been spuriously investigated. Early diagnosis and timely management of the affected infants are key to reducing bowel loss and improving the survival rate [5, 6].

In the early stage of NEC, the intestinal wall is swollen and thickened due to inflammation, and the blood perfusion of the intestinal wall is increased [7, 8]. Then, due to the continuous existence of ischemia and hypoxia of the intestinal wall, the mucosal structure of the intestinal wall is further damaged, the intestinal wall becomes thinner, and the blood perfusion of the intestinal wall is reduced or absent [9]. These pathophysiological changes might be evaluated using an abdominal ultrasound (AUS) assessment with additional detail through a real-time assessment of bowel-wall thickening, peristalsis, vascular perfusion, and other parameters [10]. Various studies have suggested that an AUS examination may be more conducive to the early diagnosis of infants with NEC than AR [11, 12]. However, no study has compared the ability of AUS and AR to predict the need for bowel resection.

Here, we intend to assess the value of AUS in predicting bowel loss in infants with NEC and compare it with AR. Identification of the potential for bowel resection among infants with NEC might optimize the perioperative care protocol to improve the clinical outcome.

## Materials and methods

### Study design and study subject recruitment

The current multicenter retrospective research protocol was approved by the institutional ethics committee at Chongqing children’s Hospital, and the requirement for consent was waived because of the retrospective design. For the purposes of this study, records of infants confirmed to have NEC between January 2018 and March 2021 were retrospectively reviewed using a comprehensive assessment, including clinical, AUS and AR evaluations. All the samples were collected from three institutes, including Chongqing Children’s Hospital, Chongqing Three Gorges Central Hospital, and the Chongqing health center for women and children. All eligible infants were identified with Bell’s stages II and III NEC, and only infants who underwent surgical intervention were included. Usually, abdominal US was performed at admission and twice daily on the second day unless the clinical condition deteriorates, prompting additional imaging. AR and at least two time points of AUS evaluation data (at admission and the day of NEC diagnosis) was available for each eligible infant. Details of all diagnoses and procedures were reviewed by the local principal investigator to make the final decision regarding inclusion. In the institutes involved, the surgical intervention methods depended on the preference of the surgical team and the disease involvement. Infants were excluded if complete data were unavailable or if death was not related to intestinal complications within postoperative Day 30.

### US machine and sonographic parameters

Abdominal AUS assessments were performed at the bedside, quadrant by quadrant, with swipe scanning in the transverse and sagittal planes. In our institute, a Ge LOGIQ e ultrasonic diagnostic instrument with an 8–18 MHz linear array probe was used by the trained sonographer. A frequency of 16 MHz was used to clearly display the intestinal wall, and the gain was adjusted to the best range. The patient was placed in the supine position, and the abdomen was examined by abdominal multisection scanning. During the examination, the most important goal is to observe the condition of the intestinal wall of the child. The abdomen was divided into four parts: left upper abdomen, right upper abdomen, left lower abdomen, and right lower abdomen. The intestinal wall thickness of each part was measured three times, and the average value was recorded. Bowel wall thinning or thickness was considered less than 1.0 mm or greater than 2.7 mm, respectively. Then, color doppler flow imaging (CDFI) was used to observe the blood perfusion of the intestinal wall, and the number of blood perfusion points and strips per unit area of intestinal wall (cm^2^) were measured. Finally, the pulsed-wave doppler (PW) mode was used to record the arterial blood flow in the intestinal wall, and the RI value of the arterial resistance index was measured. Free intraperitoneal gas was identified by the presence of linear or punctuate echogenic foci outside of the bowel with reverberation artifacts. Loss of the hypoechogenic muscle layer was regarded as hyperechogenicity. The presence of punctate or linear echogenic foci within the bowel wall and the portal vessels was characteristic of pneumatosis intestinalis and portal venous gas. The abdominal fluid was divided into three categories: simple ascites, focal fluid collections and complex ascites. Three operators completed the current evaluation with the identical procedure. The clinical data of children with NEC were collected, including gestational age, age, sex, weight, treatment course and prognosis.

### Data collection

One hundred sixty-four infants who underwent laparotomy were enrolled during the research period, and the infants were assigned to two groups based on whether intestinal resection was performed: the intestinal resection group was compared with the no intestinal resection group. The infants were dynamically evaluated using at least two AUS examinations, and we compared the changes in the former and latter parameters. The bowel wall thick to thin transformation was defined as a greater than 0.5 mm difference between the two exams. Intestinal necrosis was comprehensively evaluated based on the clinical and pathological archives, especially patients’ AUS and AR images, which determined the need for resection of the bowel segment. The AUS and AR findings were carefully reviewed by two board-certified radiologists with more than 10 years of experience who were blinded to the other diagnostic modalities and the clinical outcomes.

### Statistical analysis

The statistical software SPSS 26.0 (SPSS Corp., Chicago, IL) was utilized to analyze the data. Continuous variables with a normal distribution are presented as the means ± standard deviations and were analyzed using the unpaired Student’s t test; the abnormally distributed variables are described as the medians (ranges) and were compared utilizing the Mann–Whitney U test. Categorical variables are reported as frequencies (percentages) and were tested with the chi-square test or Fisher’s exact test for intergroup comparisons. A receiver operative characteristic (ROC) curve was analyzed to determine the area under the ROC curve (AUROC) for the ideal predictive value of the specific measurements and to assess the diagnostic performance of each AUS and AR component with respect to predicting intestinal resection. The multiple stepwise logistic regression models were constructed with AUS and AR parameters respectively (odds ratios [OR], 95% confidence interval [CI]). All statistical analyses were two-sided, with *p* < 0.05 considered significant. The goodness-of-fit was evaluated using the Hosmer–Lemeshow statistical method. ROC analyses were also conducted to compare the performances of the AUS parameters and AR parameters by comparing the AUCs of each model.

## Results

During the study interval from January 2008 to March 2021, 273 consecutive preterm infants with definite NEC who underwent surgical intervention and met the previously mentioned inclusion criteria were eligible for analysis. Among them, there were 81 babies born at the included hospitals and therefore day of admission is newborn day; there were 92 babies outborn and admission day is day of transfer day of suspected diagnosis of NEC. The detailed medical data were unable to be obtained for 83 infants, and thus they were excluded from the current analysis, and another 26 were excluded because their parents did not comply with the clinical management proposed by the physicians. Ultimately, 164 patients fulfilled the inclusion criteria and were included in the current investigation. The baseline demographic and clinical characteristics of patients stratified according to intestinal resection are reported in Table 1. The sex distribution, APGAR scores at 5  min, duration of symptoms, and enteral feeding history were comparable. No significant differences were detected in the lesion location between the two groups. The infants who underwent intestinal resection usually presented with multifocal cases or panintestinal involvement.

**Table 1:** Clinical characteristics for the infants among the two groups

	No resection (n = 68)	Bowel resection (n = 96)	*p* value
Male: female	37:31	52:44	0.55
Gestational age (week), median (IQR)	33.6 (30.1–36.7)	32.9 (29.6–36.9)	0.13
Birth weight (g), median (IQR)	2492.6 (2015.6–3253.4)	2411.8 (1989.6–3186.3)	0.22
Age at diagnosis of NEC (days), median (IQR)	15 (7–46)	11 (6–28)	0.025
No enteral nutrition before diagnosis, n (%)	7 (10.3%)	11 (11.5%)	0.52
APGAR scores at 5 min, median (IQR)	10 (9–10)	9 (9–10)	0.52
Vasopressor use at enrollment, n (%)	11 (16.2%)	15 (15.6%)	0.55
Ventilator use on day of diagnosis, n (%)	6 (8.8%)	9 (9.4%)	0.57
Acidosis, n (%)	18 (26.5%)	26 (27.1%)	0.54
Bowel involvement, n (%)			
Distal ileum	65 (95.6)	96 (100)	0.07
Distal ileum and colon	16 (23.5)	31 (32.3)	0.25
Extent of disease, n (%)			
Multifocal NEC	9 (13.2)	32 (33.3)	0.003
Localized NEC	57 (83.8)	17 (17.7)	< 0.001
Pan intestinal NEC	2 (2.9)	47 (49.0)	< 0.001

AUS and AR parameters were compared between the intestinal resection and no intestinal resection groups to identify the potential predictors of intestinal resection. As shown in Table 2, the AUS features exhibited more thick to thin transformation (OR [odds ratios], 4.38; 95% CI 2.26–8.52; *p* < 0.001), absence of certain areas of peristalsis (OR, 2.99; 95% CI 1.53–5.86; *p* = 0.001), and absence of bowel wall perfusion (OR, 10.56; 95% CI 5.09–21.90; *p* < 0.001) in the infants who underwent intestinal resection, implying intestinal necrosis, while AR revealed that portal venous gas (OR, 2.21; 95% CI 1.12–4.37; *p* = 0.016) was associated with intestinal resection. Among these characteristics, a greater number of patients presented with several parameters of AUS, which are shown in Table 2.

**Table 2:** Comparison of the AUS and AR parameters of the two groups

	No resection (68)	Bowel resection (96)	OR	95% CI	*p*
*Abdominal sonography*, n (%)					
Thick to thin transformation	22 (32.4)	65 (67.7)	4.38	2.26–8.52	< 0.001
Intramural gas (pneumatosis intestinalis)	57 (83.8)	89 (92.7)			0.063
Portal venous gas	59 (86.8)	84 (87.5)			0.54
Increased bowel wall echogenicity	24 (35.3)	91 (94.8)	33.37	11.93–93.33	< 0.001
Absent peristalsis	36 (52.9)	74 (77.1)	2.99	1.53–5.86	0.001
Simple ascites	28 (41.2)	9 (9.4)	6.77	2.92–15.66	< 0.001
Complex ascites	3 (4.4)	50 (52.1)	23.55	6.92–80.15	< 0.001
Focal fluid collections	19 (27.9)	13 (13.5)			0.019
Absence of bowel wall perfusion	18 (26.5)	76 (79.2)	10.56	5.09–21.90	< 0.001
Pneumoperitoneum	4 (5.9)	35 (36.5)	9.18	3.08–27.37	< 0.001
*Abdominal radiography*, n (%)					
Intramural gas	54 (79.4)	83 (86.5)			0.16
Portal venous gas	41 (60.3)	74 (77.1)	2.21	1.12–4.37	0.016
Dilatation and elongation	56 (82.4)	82 (85.4)			0.38
Pneumoperitoneum	6 (8.8)	43 (44.8)	8.38	3.31–21.24	< 0.001

We subsequently used the multivariable logistic models to predict intestinal resection based on the AUS parameters and AR parameters respectively. After performing the multivariable logistic regression analysis, we identified thick to thin transformation, absence of peristalsis, complex ascites, focal fluid collections, absence of perfusion, and pneumoperitoneum as independent prognostic factors associated with intestinal resection in the surgically treated infants with NEC. For AR parameters, the identified prognostic factors were portal venous gas, dilatation and elongation, and pneumoperitoneum, which were associated with intestinal resection in the infants with NEC who underwent surgery (Table 3).

**Table 3:** Multivariate logistic regression of abdominal sonography and radiography for predicting bowel resection of neonates with NEC

	Multivariate		*p*
	OR	95% CI	
*Abdominal sonography*			
Thick to thin transformation	4.21	2.02–7.94	0.0051
Absent peristalsis	2.36	1.19–4.98	0.0082
Complex ascites	12.65	4.27–39.44	< 0.001
Focal fluid collections	1.22	1.01–3.99	0.018
Absence of perfusion	3.83	1.89–13.48	0.021
Pneumoperitoneum	6.38	2.42–17.68	0.0065
*Abdominal radiography*			
Portal venous gas	1.541	1.09–3.74	0.044
Dilatation and elongation	1.19	1.00–2.43	0.027
Pneumoperitoneum	5.85	2.27–15.97	0.0034

**Fig. 1:**
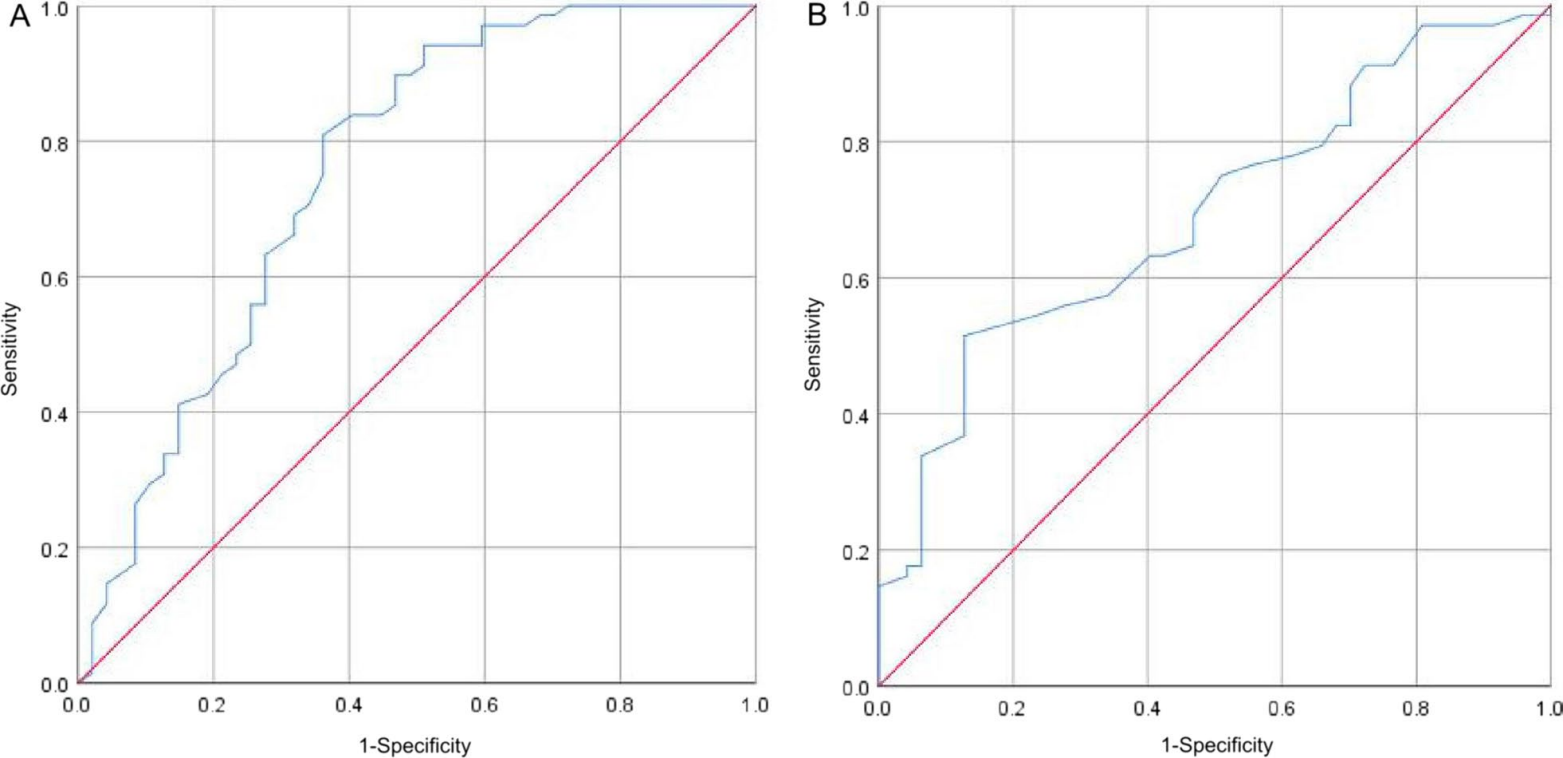
Comparison of the areas under the receiver operating characteristic curves (AUROCs) for the two logistic models. **A** Receiver operating characteristic curves for logistic models of AUS parameters in infants with necrotizing enterocolitis. **B** Receiver operating characteristic curves for logistic models of AR parameters in infants with necrotizing enterocolitis

Two ROC analyses were conducted using the six AUS parameters and three AS parameters. The ROC results for the two models are presented in Fig. 1, and the AUROC for the AUS logistic model (AUROC: 0.755, 95% CI 0.660–0.849) was significantly higher than that of the AR model (0.693, 95% CI 0.597–0.789) (*p* = 0.0021).

## Discussion

The present research furnished highly accurate ultrasonographic features for predicting intestinal resection in patients with NEC. AUS parameters were superior to the traditional AR measurement and displayed high sensitivity and specificity to detect bowel necrosis, which were associated with bowel loss. Therefore, AUS should be primarily considered during NEC evaluation due to its potential advantages over plain AR films.

Because of the rapid progress without classic features, a considerable number of infants with NEC are diagnosed at an advanced stage ultimately need bowel resection. In our study, bowel resection occurred in approximately half of the patients investigated. The current bowel resection rates (96/164, 58.5%) in infants with NEC requiring surgical management were lower than those reported previously [13–15]. Because clinical guidelines to accurately predict the timing for surgical intervention for NEC were unavailable, we preferred to make the decision regarding surgical management in a relatively early stage in our institute to avoid intestinal loss, which means that the Bell’s stages II A was the indication for the surgical intervention and account for the difference between our data and those of Western countries, though likely other factors also contribute between the difference in data between other centers. Intestinal loss should be avoided because it constitutes an urgent and important issue, which predispose infants to adverse long term nutritious and development outcome. Unfortunately, no single predictors showed clearly superior to accurately predict bowel resection in patients with NEC, when bowel ischemia or necrosis has occurred. In the current investigation, we retrospectively reviewed the clinical data from infants with NEC in terms of bowel loss and dynamically observed the intestinal wall thickness, intestinal peristalsis, and intestinal wall blood flow perfusion.

In the new diagnostic criteria of NEC that were recently revised by the Vermont Oxford Network, the specific imaging signs of an abdominal B ultrasound examination in each stage of NEC have been added [16–18]. An important meta-analysis further confirmed that ultrasonographic parameters, such as focal fluid collections, complex ascites, absent peristalsis, pneumoperitoneum, bowel wall echogenicity, bowel wall thinning, absent perfusion, bowel wall thickening and dilated bowel were predictive of death and/or surgery in neonates with NEC [19], which suggested that ultrasonography could identify high-risk infants requiring surgical intervention. In the current research, these parameters (Thick to thin transformation, Absent peristalsis, Complex ascites, Focal fluid collections, Absence of perfusion, Pneumoperitoneum) were strongly predicted the need for bowel resection.

AUS might provide an imaging reference for the selection of clinical treatment options, including description of bowel wall thickness, bowel peristalsis and perfusion, which is important for a NEC evaluation in infants with nonspecific AR findings prior to pneumoperitoneum and mild symptoms [19, 20]. Only 3 imaging findings were identified by both abdominal X-rays and ultrasound, including free air, pneumatosis intestinalis and portal venous gas. Ascites, bowel wall thickening and nonviable bowel can be insinuated from serial radiographs, but ultrasound has an obvious advantage in this regard since it can identify these findings directly [19]. Although pneumoperitoneum was detected using abdominal sonography, it is more accurately detected by X-ray than ultrasound measurements. Another point is that infants with NEC require followed and multiple repeated examinations. X-ray examination is associated with radiation, and thus it is not as suitable for long-term and multiple examinations, although abdominal XR are still widely used, especially when AUS is not as readily available. The advantages of ultrasound are non-radiation exposure, its ability to be conducted at the bedside and numerous follow-up examinations can be conducted during their hospital stay [21, 22]. These features are of critical importance for premature infants, as premature babies are particularly radiosensitive. In our current study population, the most predictive variables for bowel resection were sonographic parameters, including thick to thin transformation of the bowel wall, absence of intestinal peristalsis and blood flow perfusion loss. Feingold et al. [23] measured the intestinal wall thickness of 30 children without gastrointestinal diseases, 22 children with NEC and 8 children with suspected NEC and found that the intestinal wall of children with NEC was thickened or thinned to varying degrees, among which the intestinal wall of 8 children with severe NEC was thinned, and the follow-up found that all 8 children had intestinal necrosis. This study confirmed that the course of NEC is accompanied by a change in intestinal wall thickness, and the intestinal wall thickness in the early stage of NEC displays nonuniform thickening [24, 25]. This finding is consistent with the results of the present study showing that the intestinal wall thickness of infants with stage I and II NEC is thicker than that of infants with bowel loss. Bowel ischemia or necrosis was associated with a thinned intestinal wall. In the current study, several AR parameters were related to bowel loss, including free peritoneal gas and pneumoperitoneum. In our clinical practice, because of the use of AUS serially, AR was conducted at an early stage and seldom repeated thereafter, which account for the low detection rate of AR. Because of the subsequently high rates of bowel loss, which are characterized by the aforementioned variables, surgical consultation should be considered appropriately to treat perforated or necrotic bowel, and earlier surgical intervention was beneficial to avoid this problem, even in infants without pneumoperitoneum [20, 21]. Although most of the variables mentioned here have been previously evaluated, many of them have not been investigated in terms of bowel loss. Here, we found that the use of AUS parameters is helpful in predicting intestinal resection in infants with advanced NEC.

Several potential limitations of this study should be recognized when interpreting the current results. A weakness is that decision-making regarding bowel resection was not made randomly and only based on the experience of each surgeon. This decision is a confounder for the preference of therapy or surgeon’s personal choice with an inherent risk of selection bias. Another limitation is the retrospective design of our study. A lack of standardization of the radiographic and sonographic measurements may introduce sampling bias. Radiographic and sonographic measurements were not performed simultaneously, and not every patient underwent immediate radiographic measurements before laparotomy due to radiation exposure, which might have resulted in bias in the results. The median gestational age of infants with NEC is higher than many other cohorts so could be difficult to generalize to younger populations. Another limitation is that AUS measurements have objective components and clinicians performing the sonographic exam have varying levels of experience, and thus the results may be inaccurate. In addition, investigators were not blinded to the purpose of this study. Therefore, optimal, adequately powered studies must be performed to validate the potential utility of AUS measurement in infants undergoing bowel resection.

## Conclusion

In summary, AUS may be used in conjunction with other clinical factors to distinguish infants with the potential for bowel loss from infants with severe NEC. The AUS evaluation of NEC play an important role in rescue gastrointestinal loss during the decision to perform surgery. A future prospective study with more controlled imaging measurements would be useful to verify the current conclusions.

## Data Availability

The dataset analyzed during the current study are available from the corresponding author on reasonable request.
